# Treatment of recalcitrant hypertrophic lichen planus with upadacitinib

**DOI:** 10.1016/j.jdcr.2025.02.029

**Published:** 2025-03-12

**Authors:** Sabrina F. Schundler, Christiaan H. Noot, Zachary Frost, Jennie Clarke, Zachary H. Hopkins

**Affiliations:** a Rush Medical College, Chicago, Illinois; b Department of Dermatology, University of Utah Spencer F. Eccles School of Medicine, Salt Lake City, Utah; c Noorda College of Osteopathic Medicine, Provo, Utah

**Keywords:** HLP, hypertrophic lichen planus, JAK inhibitor, lichen planus, LP, upadacitinib

## Introduction

Hypertrophic lichen planus (HLP) is a variant of lichen planus (LP)^1^^,^^2^ that commonly involves the bilateral lower extremities and is characterized by thick hyperkeratotic nodules and plaques.^1^ HLP is often difficult to treat and can be associated with secondary squamous cell carcinoma.^1^ Treatments include high potency topical steroids, intralesional corticosteroid injections, phototherapy, and systemic agents such as prednisone, acitretin, and other immunosuppressive agents.^1^^,^^2^ Herein, we present a case of recalcitrant HLP that cleared with upadacitinib, an oral JAK inhibitor (JAKi).

## Case report

**Fig 1:**
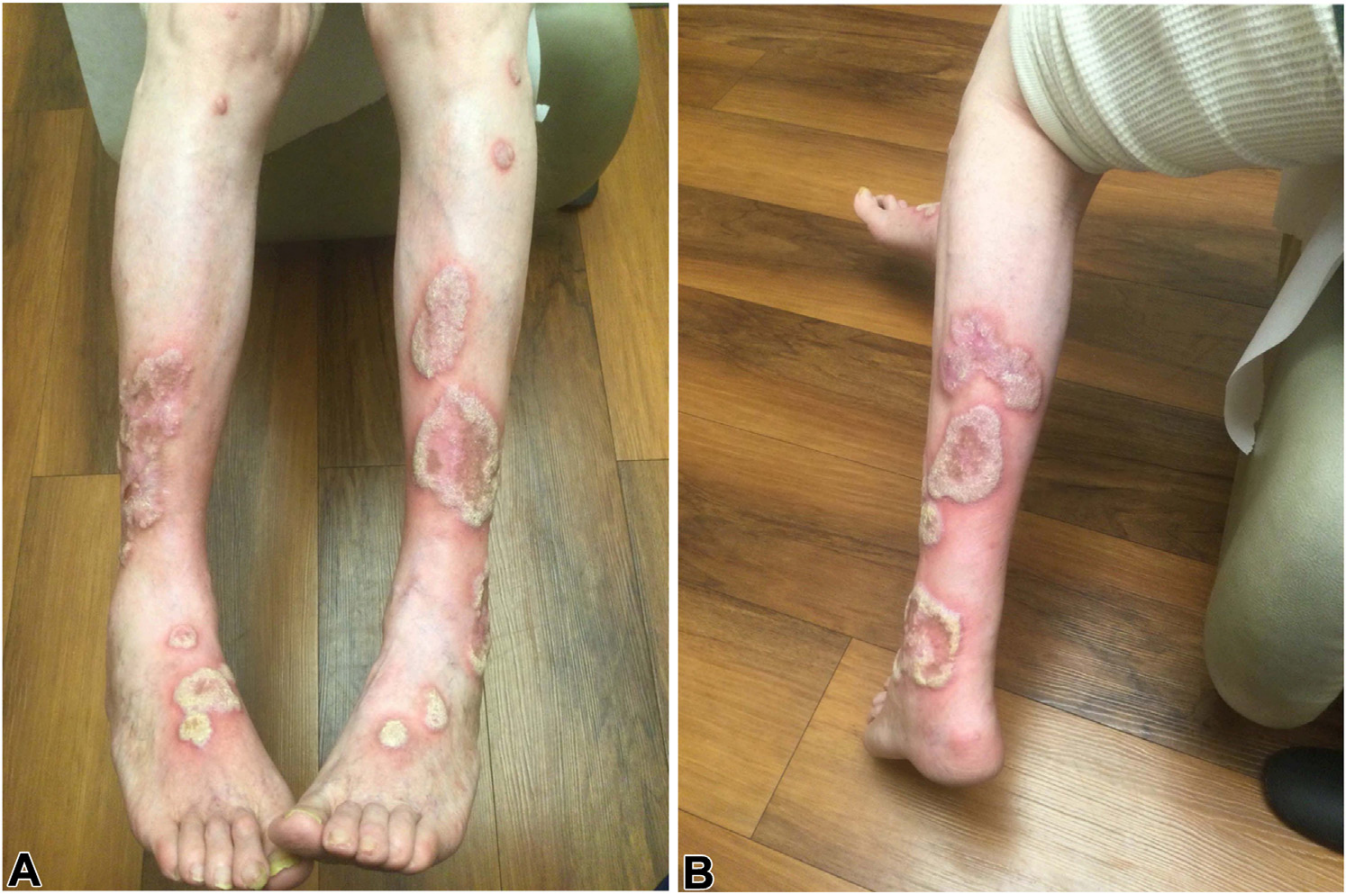
Hypertrophic lichen planus. Hypertrophic, lichenified, scaly plaques with surrounding erythema, involving the (**A**) anterior and (**B**) posterior lower portion of the legs on initial presentation.

A man in his 60s presented to our clinic with pruritic, hypertrophic, scaly plaques on the bilateral lower portion of the legs (Fig 1, *A, B*). Previous biopsies revealed band-like lymphocytic infiltrate of lymphocytes and eosinophils obscuring the dermoepidermal junction, vacuolar change, and necrotic keratinocytes consistent with a lichenoid dermatitis. Histologic features, together with clinical presentation, were suggestive of HLP. Before referral, intralesional triamcinolone, oral prednisone, narrow band UV-B, hydroxychloroquine, metronidazole, and guselkumab, were tried and were ineffective. During his first visit at our institution, cyclosporine was added to his existing regimen of hydroxychloroquine, metronidazole, prednisone, and topical clobetasol. Unfortunately, this was poorly tolerated, and methotrexate was started. The patient initially improved but then developed generalized pruritus and new-onset nodules, concerning for prurigo nodularis (PN). Biopsies of a new nodule on the forearm and a recalcitrant plaque on the leg were suggestive of PN and HLP respectively with no evidence of malignant nor infectious etiologies contributing to the recalcitrant HLP plaques on the legs. We then initiated Unna-boots with topical clobetasol for his HLP and dupilumab for his PN.

**Fig 2:**
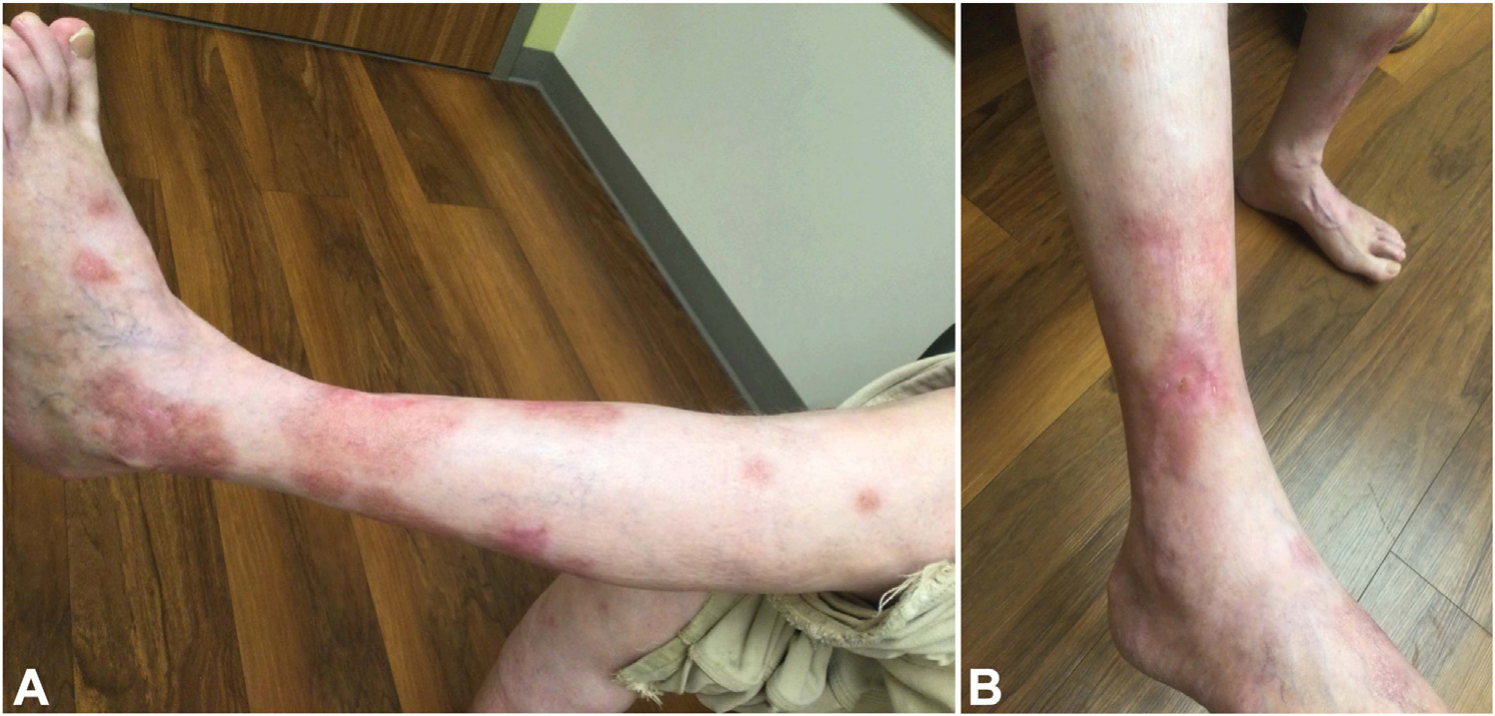
Postinflammatory hyperpigmentation and atrophy. Complete resolution of HLP lesions of the (**A**) anterior aspect of the left leg and (**B**) lateral aspect of the right leg.

After 12 months his pruritus and prurigo nodules improved, but the HLP continued with some worsening. During this time, samples of topical ruxolitinib were provided, but were ineffective. Given the continued severe impact on the patient’s quality of life, we started upadacitinib 15 mg daily. After 1 month his HLP was nearly clear (Fig 2, *A, B*). Notably, the patient reported complete resolution of itch within 1 week. After 3 months, the dupilumab was discontinued. The patient continues on upadacitinib 15 mg daily, maintaining complete clearance of the HLP and PN for 1 year with no reported side effects.

## Discussion

CD8^+^ T cell-mediated immune responses targeting basal keratinocytes are believed to play a central role in HLP.^2^ This process appears to be primarily T-helper types 2 and 17-driven, with elevated levels of interferon gamma indicating a role for the Janus kinase-signal transducer and activator of transcription pathway (especially JAK1/2).^3^ Additionally, JAK2 inhibition has been shown to protect keratinocytes from cytotoxic responses.^4^ Accordingly, there has been increasing interest in JAKi use for LP.

Data supporting use of JAKi use in LP remains sparse. A phase 2 clinical trial of topical ruxolitinib showed significant improvement in LP lesion counts and severity^5^ and a multicenter phase 3 clinical trial (ClinicalTrials.gov, NCT05593432) is currently nearing completion. Topical ruxolitinib was trialed for our patient but failed to improve his HLP—perhaps stemming from the thickness of the lesions. Research on systemic JAKi use in LP is highly limited. In a recent review of systemic JAKi therapy for different forms of LP, tofacitinib, a potent JAK1/3 inhibitor was most reported (8 reports), followed by baricitinib, a selective JAK1/2 inhibitor (5 reports), and upadacitinib, a selective JAK1 inhibitor (2 reports). Most reports with tofacitinib were in lichen planopilaris and showed mixed results (60% had partial improvement and 10% achieved complete resolution).^4^ In the 2 case reports of upadacitinib, its use led to complete resolution of erosive oral LP.^3^^,^^4^

Although upadacitinib is a selective JAK1 inhibitor, one study demonstrated pronounced impact on JAK1 and JAK2 signaling pathways with therapeutic doses, including more potent inhibition of JAK2-dependent cytokines interleukin 3 and granulocyte-macrophage colony-stimulating factor than tofacitinib and baricitinib.^6^ This broad JAK1/2 inhibition may provide efficacy in LP. Further data are needed to fully elucidate in vivo JAKi effects on LP and prospective studies are needed assessing their efficacy and safety.

This case highlights the potential use of JAKi therapy for recalcitrant HLP. This is especially salient since HLP is often challenging difficult to treat and can be associated with secondary squamous cell carcinoma. Further investigation is needed for JAKi-based therapy in lichenoid dermatoses.

## Conflicts of interest

None disclosed.
